# Microstructure and Selected Properties of Cr_3_C_2_–NiCr Coatings Obtained by HVOF on Magnesium Alloy Substrates

**DOI:** 10.3390/ma13122775

**Published:** 2020-06-18

**Authors:** Ewa Jonda, Leszek Łatka, Wojciech Pakieła

**Affiliations:** 1 Department of Engineering Materials and Biomaterials, Silesian University of Technology, Konarskiego St. 18a, 44-100 Gliwice, Poland; wojciech.pakiela@polsl.pl; 2 Department of Metal Forming, Welding and Metrology, Faculty of Mechanical Engineering, Wroclaw University of Science and Technology, Łukasiewicza St. 5, 50-371 Wroclaw, Poland; leszek.latka@pwr.edu.pl

**Keywords:** Cr_3_C_2_–NiCr cermet powder, HVOF spraying, magnesium AZ31 alloy, microstructure, instrumented indentation, erosion resistance

## Abstract

In present work the Cr_3_C_2_–NiCr coating was deposited on magnesium alloy substrate with high velocity oxygen fuel (HVOF) spraying. The microstructure of the samples has been characterized by means of electron microscopy, SEM and phase composition analysis carried out. The porosity of coatings has been also estimated. Finally, tests of selected mechanical properties, such as instrumented indentation, abrasive erosion have been performed. The results of the investigations confirmed that dense, homogeneous and well-adhered Cr_3_C_2_–NiCr cermet coating is possible to obtain onto the magnesium AZ31 alloy substrate. Moreover, the coatings exhibit high resistance to erosion.

## 1. Introduction

For several years, magnesium alloys have been the subject of research in numerous scientific and research centers, as well as among industry producers, including automotive, machine construction, shipbuilding, aviation, chemical, energy, electronic and textile industries. Their low density (1.5–1.8 g/cm^3^), the best among all currently known construction materials and high strength in relation to low weight, make these alloys able to be seen as the material of the future. However, low resistance to corrosion, erosion and abrasive wear can adversely affect and limit their widespread use [1,2,3,4,5,6]. The solution of this problem is to create protective layers, among others coatings applied by thermal spraying methods, which are used to extend the durability and life of machine parts, both new and remanufactured. Depending on the type of heat source used to melt the coating material, spraying can be distinguished: flame (low and high velocity), detonation, arc and plasma. The feedstock material could have a form of powder or wire. The most commonly used methods among above mentioned ones are the high velocity oxygen fuel (HVOF) and atmospheric plasma spraying (APS). The HVOF method allows the production of coatings with special properties, among others: low oxidation, very low porosity and high adhesion strength. Important advantage of this method is manufacturing coatings, which exhibit high abrasion resistance, protection against high temperature, erosion and corrosion. The wide variety of the feeding materials chemical composition makes it possible to produce coatings with dedicated properties for specific applications [7,8,9,10].

**Figure 1:**
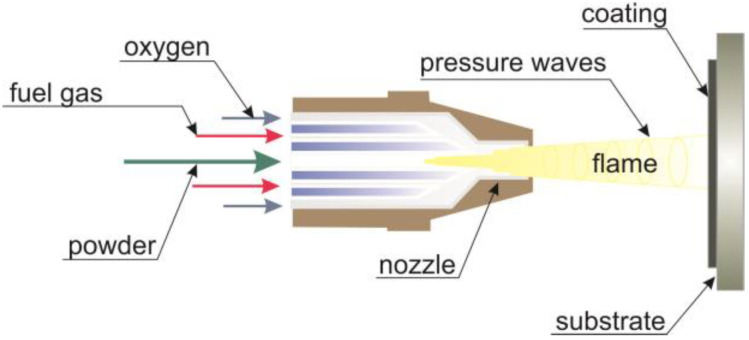
Scheme of the high velocity oxygen fuel (HVOF) spraying process, adapted from [8].

In the HVOF (high velocity oxygen fuel) process, molten or partially molten material particles are sprayed onto the substrate surface with a high-speed, high-temperature gas stream. The coating material is heated and accelerated with a gas stream jet, and then hits the surface of the deposited element. In this process, the gas stream is produced by mixing and igniting oxygen and fuel (gaseous or liquid) in the combustion chamber, ensuring a rapid flow of gas under high pressure through the nozzle. The velocity of the gas stream that lifts the particles is supersonic. Particles of the coating material are introduced into the stream, heated and directed to the surface of the element. The mixture of oxygen and fuel creates high pressure in the combustion chamber, and then passing through the expanded Laval nozzle forms a shock wave. Gases that explode inside the shockwave melt the powder particles and transport it towards the substrate on which the coating is formed, see Figure 1 [11,12,13,14,15].

**Figure 2:**
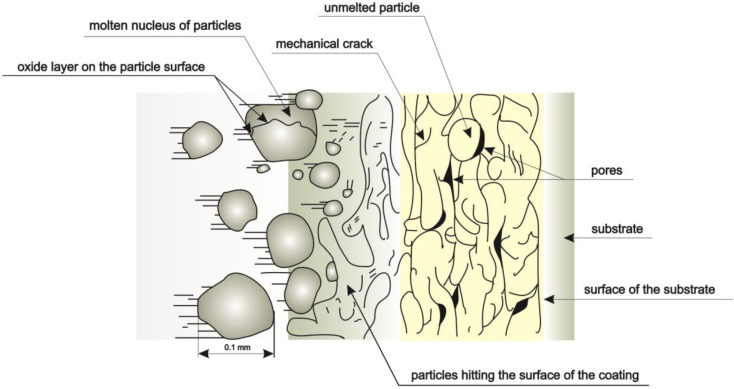
Scheme of the coating formation, adapted from [20].

In the spraying process, the substrate material does not melt, and the sprayed coating is attached to the substrate mechanically, with adhesion or, in special cases, the connection is of metallurgical nature. Particles of material falling on the sprayed surface in a semi-plastic state are flattened and, adhering to each other in the form of successive layers, form a continuous coating. (Figure 2, scheme of the coating formation) [16,17,18,19].

The analysis of current research results in the field of coating production by means of supersonic flame spraying (HVOF) includes spraying, among others on alloy structural steels, non-alloy qualitative steels for thermal improvement, stainless and on nickel alloys, while the use of light construction materials as foundations in the form of magnesium alloys with low resistance to tribological factors has not been thoroughly studied and discussed so far.

Review of the presented results of experimental works indicates that in the field of development of modern techniques for improving or restoring the properties of the surface of an element by supersonic spraying, there are few studies on research works on the production of the abovementioned coatings on magnesium alloys [21,22,23,24,25].

In spite of plenty of research, there is still not enough information about the microstructure and properties of the Cr_3_C_2_–NiCr coatings obtained by HVOF on magnesium alloy substrates. This manuscript is going to be an attempt to fill this gap as a current topic, both from a scientific and application point of view.

In this publication, the authors focused on preliminary studies, which aim was check the possibility of manufacturing cermet coating using HVOF method onto AZ31 magnesium alloy. Additionally, these coatings should be characterized by good adhesion, low porosity and proper tribological properties. Therefore, the article presents and discusses the results only for one Cr_3_C_2_–NiCr coating obtained with initially optimized parameters by CERTECH Company, Wilamowice, Poland. In future research, authors will continue the optimization process for this powder in order to improve functional properties of obtained coatings.

## 2. Materials and Methods

### 2.1. Coatings Deposition

AZ31 magnesium alloy was selected as a substrate material, with the chemical composition: (elements, in wt %) Mn—0.17, Zn—1; Al—3, Ca—0.04, Cu—0.05 and Mg—balance, with 5 mm in the thickness (Institute of Non-Ferrous Metals in Gliwice, Skawina, Poland) [26]. The particle size of commercially available powder Cr_3_C_2_–25% wt. NiCr (Amperit 588.059) was in the range of 30 + 5 μm and supplied by Höganäs. The chemical composition of the feedstock powder, which was used in coatings manufacturing process is (elements, in wt %): Cr: 66–73, C: 9–11, Fe: <0.5, Ni: 15–22 and O: <0.6 [27].

The surface of samples was prepared by sandblasting with corundum to get a surface roughness in the range of Ra equal to 17 μm and ultrasonic treated to achieve god adhesion between coating and the substrate. C-CJS spray system Thermico (CERTECH Company, Wilamowice, Poland) was used to prepare coating. Kerosene and oxygen were used as the fuel gases, respectively, whereas nitrogen was used as the carrier gas. The maximum flame temperature with in the hottest point was about 3520 K, whereas the substrate surface temperature, including spray distance (280 mm) did not exceed 600 K. The spraying parameters are listed in Table 1.

### 2.2. Coatings Characterization

#### 2.2.1. The Microstructure

Microstructural investigations were carried out by means of scanning electron microscopy, SEM (Supra 35, Zeiss, Oberkochen, Germany) using secondary electron and backscattered detectors (TRIDENT XM4, Edax, 91 McKee DRIVE, Mahwah, NJ, USA). The chemical composition was analyzed by EDS (energy dispersive X-ray spectroscopy, Supra 35, Zeiss, Oberkochen, Germany). In order to determine the phase composition of the Cr_3_C_2_–NiCr coating, X-ray diffraction, XRD, tests were carried out. XRD investigations of sprayed coatings were done by an X-ray diffractometer X’Pert Pro MPD by Panalytical (Almelo, the Netherlands) apparatus with a copper anode lamp (λKα = 0.154 nm; Panalytical, Almelo, the Netherlands) as well as a PIXcel 3D detector (Panalytical, Almelo, the Netherlands) on the diffracted beam axis. The diffraction lines were recorded in the Bragg–Brentano geometry in the angular scope of 15°–90°, with the step of 0.03° and the step time of 0.8 s. The analysis of the obtained diffraction patterns was made in the Panalytical High Score Plus software (Version 3.0e, Panalytical, Almelo, the Netherlands), containing a dedicated flat-file base of PAN-ICSD phase identification. The coating’s surfaces observation was carried out with digital optical microscope Keyence VHX-5000 (Keyence International, Mechelen, Belgium). The surface morphology of the sprayed coating was examined using an atomic force microscope (AFM; XE-100 Park Systems Corp, Suwon, Korea).

#### 2.2.2. Surface Topography, Porosity and Roughness

Measurements of surface topography were carried out under ambient conditions and with the use of a commercial scanning probe. The AFM topographic images were analyzed with the use of a dedicated XEI program (Version of XEI program-1.08, Park Systems Corp., Suwon, Korea). Using AFM microscopy (100 Park System, Park Systems Corp., Suwon, Korea), Ra (arithmetic mean of ordinates of the roughness profile) and Rz (maximum height of the roughness profile) roughness parameters were made. The sandblasting was used in order to increase the surface roughness, which is necessary to improve the coatings adhesion strength to the substrate. Image analysis with free software ImageJ (version 1-50) was used to determine the porosity level. The measurements were made according to the ASTM E2109-01 standard [28]. The porosity was assessed as an average from 20 images at 1000× magnification.

#### 2.2.3. Mechanical Properties

The testing of mechanical properties was started by measurements of coatings adhesion strength. The bond-strength was determined by a well-known pull-off test and was carried out by a dedicated Elcometer 510 tester (Elcometer Instruments, Manchester, UK). A counter-part with the diameter of 10 mm was bonded to the coating using epoxy adhesive Distal Classic (Libella, Warsaw, Poland). Then, the continuous pressure of 0.1 MPa/s was applied to the test dolly. After the observations of coating surface and dolly surface, the failure mode was determined. The average value and standard deviation were calculated based on four measurements. Hardness of manufactured coatings was determined by an instrumented indentation test (IIT). Tests were carried out on an NHT^3^ nanoindenter (Anton Paar, Graz, Austria), equipped with Berkovich indenter and were performed at room temperature on the cross sections in accordance with ISO 14577-4:2016 standard [29]. With this method, also an instrumented Young modulus of sprayed coating was determined, based on Oliver and Pharr methodology [30]. The details of the measurement procedure could be found in [31]. For hardness measurements, the value of the maximum load was equal to 500 mN, whereas for elastic modulus the range of maximum load was from 50 to 500 mN. In both cases the dwell time was equal to 15 s.

#### 2.2.4. The Abrasion and Erosion Resistance

Tests of erosion resistance at room temperature were carried out at a specially constructed stand at the Welding Department of the Silesian University of Technology in Gliwice, according to the ASTM G76-04 standard [32]. The test used a nozzle tube with a diameter of 1.5 ± 0.075 mm located at a distance of 10 ± 1 mm from the sample, the angle of inclination of the nozzle in relation to the sample was 90°. The tests were carried out using Al_2_O_3_ abrasive with a grain diameter of 50 µm, particle feed rate of 2 ± 0.5 g/min and discharge velocity from the nozzle 70 m/s. The test duration was 5 min and was determined experimentally because at 10 min the erosion crater was deeper than 1 mm. In order to obtain reliable results, 10 tests were carried out with given parameters. Due to the very small weight losses bordering the measurement error, the erosion crater profiles were measured (digital optical microscope Keyence VHX-5000) and observed by the scanning electron microscope (SEM, Supra 35, Zeiss, Oberkochen, Germany). In addition to the erosion resistance tests, abrasion resistance tests were also performed. The abrasion resistance tests were carried out using the “pin-on-disc” method in accordance to the ASTM G99 standard [33]. The ZrO_2_ (zirconium oxide) ball with a diameter of 6 mm was used as a counter-body. The tests were carried out at room temperature, with the following parameters: load—5 N, linear speed—20 cm/s and distance—500 m. During the test, the friction coefficient was measured. To determine the mechanism of the wear the topography was analyzed using an SEM and the wear track dimensions after tests were measured by a Sutronic 25-Taylor Hobson profilometer (Taylor Hobson Ltd., Leicester, England).

## 3. Results

### 3.1. The Microstructure

**Figure 3:**
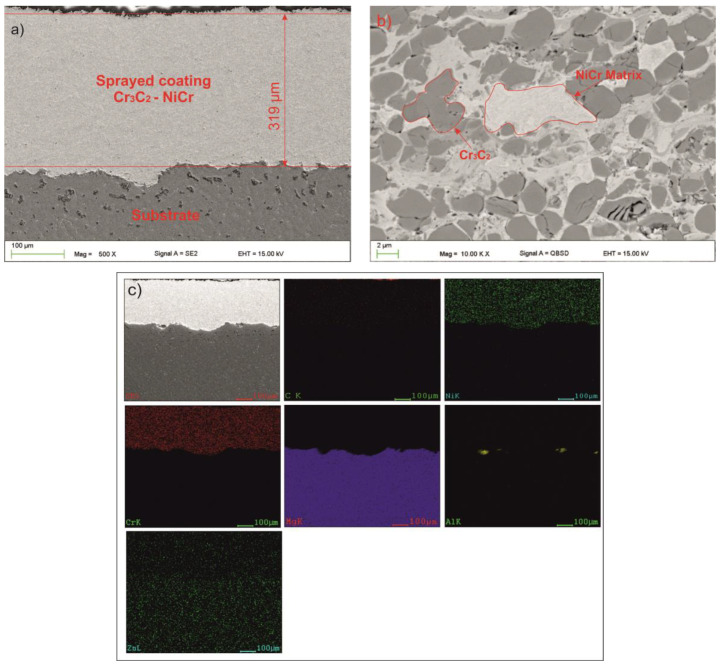
Structure of the coating (**a**) thickness; (**b**) central part of the layer and (**c**) elemental distribution maps of spraying elements in the analyzed area of the coating obtained during thermal spraying.

The structure observation in a scanning electron microscope was made in BSD (backscattered electrons) and SE (secondary electrons) mode. In the area of the layer did not disclose cracks or voids. The porosity level of the coating was equal to 2% ± 0.3% (Figure 3a,b). The occurrence of cracks and voids could result in reduced durability of the coating, as well as, crack propagation and finally delamination [34]. SEM micrographs of the coating have indicated unmelted carbide particles (dark areas on the Figure 3b) in the metallic (Ni–Cr) matrix. Size of the carbides in the layer did not exceed 3 μm. In addition, their shape (rounded edges) clearly indicates that they were melted during the HVOF process. Cr_3_C_2_ carbides were evenly distributed in the metal matrix (Figure 3b,c). Evenly distributed structure components, including carbides, significantly affect the strength of the coating. Sidhu et al. has come to similar conclusions. They combined the increase in hardness of the obtained coatings with a high volume of carbides that were well-dispersed in the matrix [35]. The shape and distribution of carbides in the matrix indicate correctly selected process parameters.

**Figure 4:**
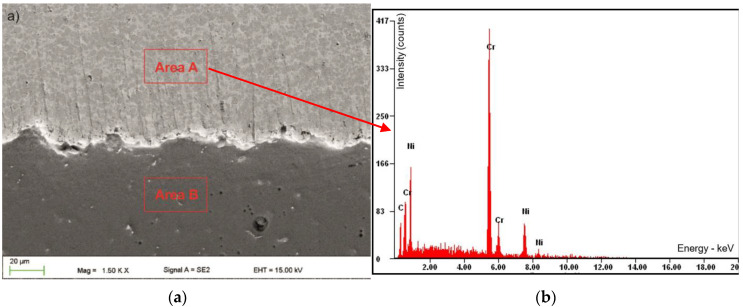
SEM cross-section images: area “A” coating and area “B” substrate (**a**) and the (**b**) EDS analysis of the coating.

The average coating thickness ranged from 330 ± 15 µm. J.M. Guilemany et al. showed that the thickness of the thermal spray coating has a significant impact on the quality of the electrochemical barrier. They proved that the thickness of the coating should be at least 300 µm [36] (Figure 3a). Observations of the lower part of the layer showed that the connection is only adhesive and mechanical (Figure 4a). The layer adheres tightly to the substrate. The EDS analysis showed no evidence of a diffusion connection. Carbon in the chemical composition analysis should be treated only as a guide (Table 2).

**Figure 5:**
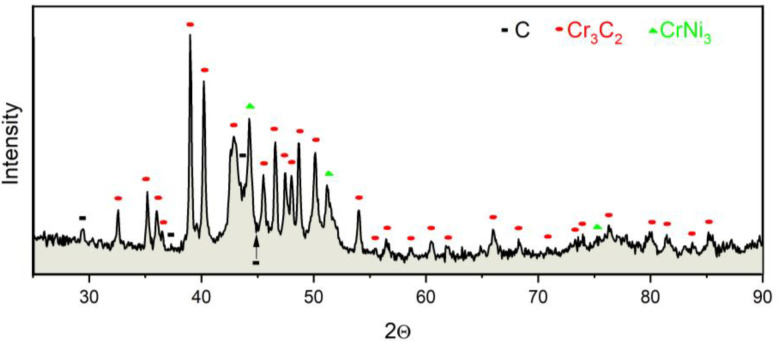
The phase composition of manufactured coating Cr_3_C_2_–NiCr deposited on AZ31 magnesium alloy.

In Figure 5 the X-ray diffraction pattern of manufactured coating is presented. It could be found two main phases: (i) an adhesive CrNi_3_, which gives a high bonding strength as well as fracture toughness and (ii) a hard Cr_3_C_2_, which provides higher hardness and wear resistance. These phases are also detected in [37]. In order to determine quantitative phase composition, the RIR (Reference Intensity Ratio) method has been used [38]. The results of these analyses are as follows: 65% of the Cr_3_C_2_ and 35% of the CrNi_3_. These values are confirmed by [39,40].

### 3.2. The Topography and Roughness of Coating

**Figure 6:**
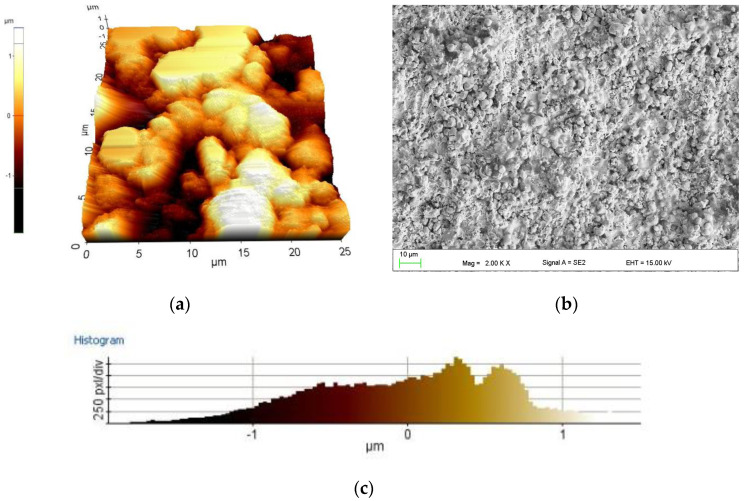
Topography of the thermally sprayed coating surface AFM (**a**) and SEM (**b**); roughness histogram over the entire surface of the coating (**c**).

The surface roughness parameters of the manufactured coating are collected in Table 3. The results were the average values from five measurements. Figure 6a,b shows the topography and (c) the roughness histogram over the entire surface of the coating. Similar values of the roughness parameters were observed for a medium carbon steel equivalent AISI4043 thermally sprayed WC–Co–Cr by HVOF [41] and also for part of the worn shafts of THM gas turbine engines with coatings of Cr_3_C_2_–25%NiCr and WC–12%Co powders carried out by thermal spray with supersonic flame (HVOF) [42]. The adhesive strength of HVOF Cr_3_C_2_–NiCr coating was from 42 to 44 MPa. The similar results (25–40 MPa) are shown by Wang et al. for WC–Co coating sprayed by HVOF on mild steel [43].

### 3.3. Wear Resistance

**Figure 7:**
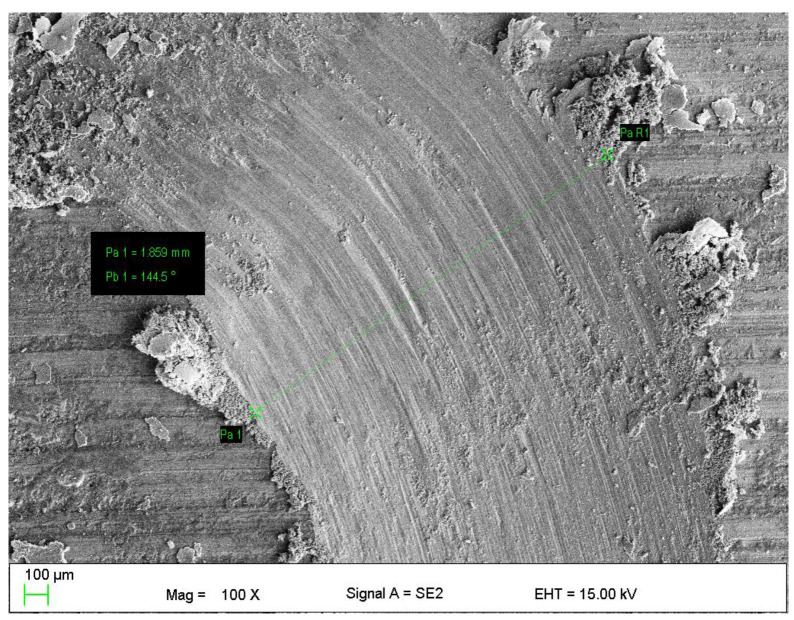
Wear tracks of Cr_3_C_2_–NiCr coatings, magnification 100×.

Manufactured coating has good wear resistance as shown by the dry sliding wear test. The wear rate of investigated coatings was equal to 13.88 ± 3.11 × 10^−6^ mm^3^/(N·m) and friction coefficient was equal to 0.6 ± 0.03. The typical wear trace is given in Figure 7. More details could be found in [44].

### 3.4. The Instrumented Indentation

The instrumented indentation tests have been carried out in order to determine microhardness and elastic modulus. In the case of hardness, the estimated value was equal to 8.7 ± 0.5 GPa and it was an average of 10 measurements. As it can be seen, the standard deviation value was quite small, which indicates low porosity and a homogenous structure (which is observed in Figure 6). Similar values for Cr_3_C_2_–NiCr coatings could be found in [37,45,46,47].

As it was mentioned above, from the unloading part of the indentation curve it is possible to determine the elastic modulus. For this purpose 10 indentation curves with the maximum load in range from 50 to 500 mN (with a step equal to 50 mN) were analyzed. The obtain value of the instrumented elastic modulus was equal to 224 GPa. This value was about 60% of the Young modulus for bulk material [48]. In comparison with another result in literature, the obtained value was similar. Slight differences could result in a different structure (mainly the porosity level) and details of the phase composition, which is a derivative of the process parameters [49,50].

### 3.5. The Erosion Resistance

**Figure 8:**
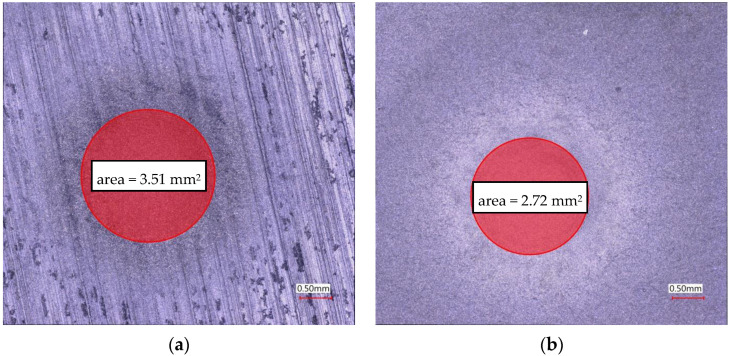
The comparison of the erosion craters of: (**a**) magnesium substrate and (**b**) composite coating.

The results of erosion test confirmed, that application of Cr_3_C_2_–NiCr composite coating strongly improves resistance against eroded particles. The erosion craters on the surfaces of the pure magnesium alloy substrate and covered by composite coating are presented in Figure 8. As it could be seen, the reduction of the erosion crater area was almost 25% for the coated surface.

**Figure 9:**
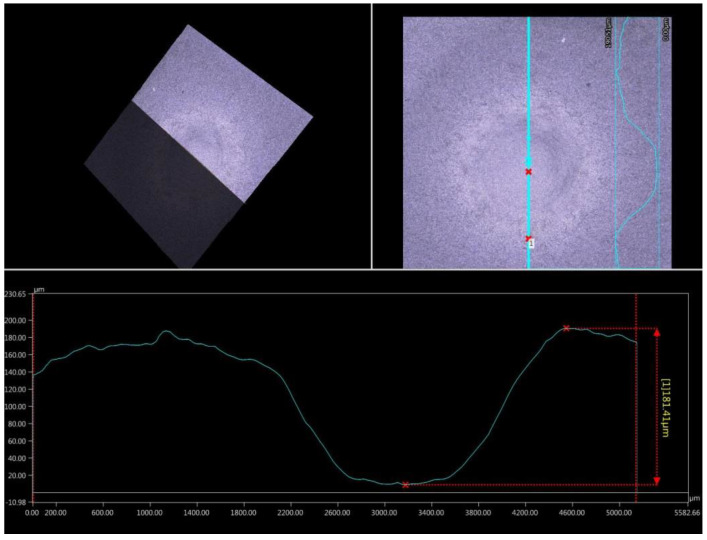
The virtual cross section of the crater of coated sample and its depth.

Moreover, the virtual cross section made in Keyence VHX-5000 software allows one to estimate the crater depth. The measurement carried out in two perpendicular directions. The view of typical virtual cross section is presented in Figure 9. Detailed values of crater depth for both samples are collected in Table 4.

**Figure 10:**
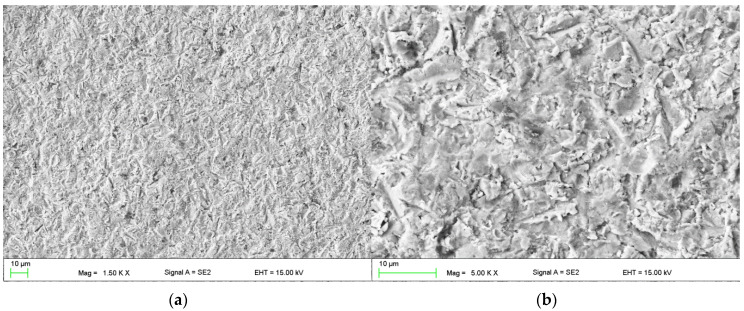
Coatings surface after erosion test: (**a**) lower magnification (1500×) and (**b**) higher magnification (5000×).

The calculated relative weight loss was equal to 0.054% ± 0.005% and 0.013% ± 0.001% for the uncoated and coated sample, respectively. The value for Cr_3_C_2_–NiCr coating was similar to that obtained by researchers in [51]. The typical surface of the coating after erosion is presented in Figure 10.

The observation of eroded surface revealed the existing of the cracks, which were initiated in the weak cohesion areas and then propagated along the interfaces between the lamellae. Additionally, the ploughing was visible [51]. The cracks exhibited a brittle type, despite the presence of ductile NiCr matrix. The occurred erosion mechanism is similar to the one observed and described by Matthews et al. [39].

## 4. Conclusions

Based on the tests carried out on the Cr_3_C_2_–NiCr coating deposited on the AZ31 magnesium alloy substrate with high velocity oxygen fuel (HVOF) spraying, the following conclusions could be made:

1. In the area of the coating did not disclose cracks or voids. The standard deviation value was quite small, which indicates on low porosity and homogenous structure (the porosity of the coating was around 2%). SEM micrographs of the coating have indicated unmelted carbide particles in metallic (Ni–Cr) matrix. Cr_3_C_2_ carbides were evenly distributed in the metal matrix. In the sprayed coatings are two main phases, namely Cr_3_C_2_ and CrNi_3_.
2. The average coating thickness ranged from 330 ± 15 µm and the coating adhered tightly to the substrate. EDS analysis showed no evidence of a diffusion connection.
3. The roughness of the sprayed coating was greater than the roughness of the AZ31 magnesium alloy substrate (Ra = 0.31–0.4 µm) and the adhesive strength of coating was from 42 to 44 MPa.
4. Manufactured coating had good wear resistance and the dominant mechanism of wear was a classic adhesive one.
5. Application of the Cr_3_C_2_–NiCr composite coating strongly improve resistance against eroded particles. The calculated relative weight loss was equal to 0.054% and 0.013% for uncoated and coated sample, respectively.

## Figures and Tables

**Table 1:** Process parameters of HVOF coating.

Coating Process	Cr_3_C_2_–NiCr
Kerosene flow rate, l/min	40
Oxygen flow rate, l/min	35
Nitrogen flow rate, l/min	10
Powder feed rate, g/min	25
Spray distance, mm	280

**Table 2:** Chemical composition of layer and substrate from the Figure 5.

Area “A” from Figure 5			Area “B” from Figure 5		
Element	wt %	At%	Element	wt %	At%
C	11	35	Mg	94	95
Cr	60	45	Al	03	02
Ni	29	20	Zn	03	02
Matrix	Correction	ZAF *	Matrix	Correction	ZAF

* ZAF (Atomic number “Z”, Absorption effect “A” and Fluorescence effect “F”)-corrections used to EDS measurements to convert raw peak intensity into semi-quantitative concentrations corrected for inter-element matrix effects.

**Table 3:** The average surface roughness of the coating in the as-sprayed condition.

Roughness Parameter	Ra (Arithmetic Mean of Ordinates of the Roughness Profile)	Rz (Maximum Height of the Roughness Profile)
Surface roughness values, μm	1.368	8.813
Standard deviation	0.126	0.619

**Table 4:** The comparison of the crater depth for the uncoated and coated sample.

Direction of Virtual Cross Section	Horizontal	Vertical	Standard Deviation	
			Horizontal	Vertical
Uncoated AZ31 magnesium alloy	286.39 µm	249.88 µm	28.53	24.21
Coated AZ31 magnesium alloy	204.32 µm	181.41 µm	16.36	15.34
